# Supplementary material for: The Timing of Water and Beverage Consumption During the Day Among Children and Adults in the United States: Analyses of NHANES 2011–2016 Data
Source: Nutrients. 2019 Nov 8;11(11):2707. doi: 10.3390/nu11112707 (PMC6893716; doi:10.3390/nu11112707)
Supplement: Supplementary file 1 [file nutrients-11-02707-s001.pdf]

nutrients

MDPI

Table S1. Water intakes (mL/d) from beverage categories (Mean, standard error).

	Milk	Milk substitutes	Citrus juices	Non citrus juices	Soda	Diet soda	RTD tea	RTD coffee	Fruit drinks	Sport drinks	Energy drinks	Hot coffee/tea	Alcohol bev	Flavored water	Other bev	Suppl
All (22,716)	79(2)	3(<1)	30(1)	25(1)	149(3)	77(3)	108 (3)	12 (1)	54(2)	30(2)	8(1)	282 (5)	129 (4)	20 (1)	16(1)	12(1)
Sex																
Men (*N* = 11,206)	95(4)	3(0.4)	36(3)	26(1)	188(6)	79(5)	116(7)	10(2)	60(3)	46(4)	13(1)	297(11)	197(11)	19(2)	17(2)	14(2)
Women (*N* = 11,510)	64(3)	3(0.3)	23(1)	25(1)	112(5)	75(5)	101(6)	13(1)	48(2)	15(2)	3(0.44)	268(9)	63(3)	22(2)	15(1)	10(1)
test	<.0001	0.5473	<.0001	0.2917	<.0001	0.5305	0.0445	0.2128	0.0002	<.0001	<.0001	0.0011	<.0001	0.095	0.39	0.0913
Age																
4–8 y (*N* = 2644)	197(8)	4(1)	29(3)	64(4)	61(5)	4(1)	23(4)	0(0)	85(4)	19(3)	0(0)	4(1)	0(0)	8(2)	7(2)	3(1)
9–13 y (*N* = 2501)	150(7)	3(1)	30(3)	33(3)	126(6)	14(2)	48(5)	3(1)	87(5)	39(5)	0(0)	11(2)	0(0)	7(2)	14(2)	3(1)
14–18 y (*N* =2308)	126(7)	2(1)	30(2)	33(3)	190(9)	21(6)	90(10)	9(2)	90(11)	52(10)	8(2)	39(6)	19(8)	17(6)	10(2)	7(2)
19–30 y (N = 3248)	57(5)	3(1)	34(3)	28(3)	220(8)	45(6)	96(7)	19(3)	68(5)	51(6)	19(3)	161(11)	172(13)	16(2)	15(2)	20(3)
31–50 y (*N* = 5071)	54(3)	3(0.4)	28(2)	18(1)	195(11)	105(10)	123(9)	13(2)	46(3)	33(4)	11(2)	333(12)	195(12)	28(4)	17(2)	14(2)
51–70 y (*N* = 4873)	60(4)	3(0.5)	24(2)	19(2)	103(6)	126(9)	148(11)	13(3)	33(3)	15(3)	4(1)	483(17)	152(11)	27(3)	18(3)	10(2)
>70 y (*N* = 2071)	71(5)	3(1)	37(2)	19(2)	51(4)	62(5)	98(8)	7(1)	29(2)	7(2)	0(0)	412(14)	61(5)	9(2)	17(3)	12(1)
test	<0.0001	0.4774	0.0011	<0.0001	<0.0001	<0.0001	<0.0001	<0.0001	<0.0001	<0.0001	<0.0001	<0.0001	<0.0001	<0.0001	0.0005	<0.0001
Time of Consumption (h)																
06:00–09:00	15(1)	1(0.1)	11(1)	4(0.3)	8(1)	7(1)	9(1)	4(1)	5(1)	3(1)	2(0.3)	135(5)	1(1)	2(0.4)	4(0.5)	4(0.4)
09:00–12:00	15(1)	1(0.1)	8(0.5)	5(0.3)	21(1)	14(1)	19(2)	3(0.4)	8(0.5)	5(1)	2(0.3)	64(2)	6(2)	4(1)	3(0.2)	3(0.3)
12:00–15:00	11(1)	0(0.1)	2(0.2)	4(0.3)	31(1)	18(1)	25(2)	1(0.1)	9(1)	6(1)	1(0.4)	15(1)	10(1)	4(0.6)	2(0.2)	1(0.3)
15:00–18:00	5(0.4)	0(0)	2(0.2)	4(0.4)	27(1)	13(1)	16(1)	2(0.3)	10(1)	6(1)	1(0.2)	15(1)	20(1)	3(0.3)	2(0.2)	1(0.2)
18:00–21:00	17(1)	1(0.1)	2(0.2)	5(0.3)	37(1)	15(1)	27(2)	1(0.2)	13(1)	5(1)	1(0.2)	15(1)	53(3)	4(1)	2(0.2)	1(0.2)
21:00–00:00	10(1)	0(0)	2(0.2)	2(0.2)	16(1)	7(1)	8(1)	0(0)	5(0.3)	3(0.4)	0(0)	8(1)	27(2)	2(0.3)	1(0.1)	1(0.2)
00:00–06:00	7(0.4)	0(0)	2(0.3)	2(0.2)	9(1)	3(0.4)	5(1)	1(0.2)	3(0.3)	1(0.2)	1(0.1)	30(2)	11(1)	1(0.3)	1(0.5)	1(0.3)
test	<0.0001	<0.0001	<0.0001	<0.0001	<0.0001	<0.0001	<0.0001	<0.0001	<0.0001	<0.0001	<0.0001	<0.0001	<0.0001	<0.0001	<0.0001	<0.0001
Type of Meal																
Breakfast	22(1)	1(0.1)	18(1)	6(0.3)	9(1)	6(1)	8(1)	5(0.4)	7(0.4)	2(0.3)	2(0.3)	149(4)	1(0.2)	1(0.2)	6(1)	5(0.5)
AM Snack	4(0.4)	0(0)	2(0.3)	3(0.3)	16(2)	13(1)	17(2)	3(1)	5(1)	6(1)	2(0.3)	76(5)	10(2)	5(1)	1(0.2)	1(0.2)
Lunch	16(1)	0(0)	3(0.3)	5(0.3)	42(2)	21(1)	29(2)	1(0.2)	12(1)	5(0.5)	1(0.3)	14(1)	5(1)	4(0.4)	3(0.3)	2(0.2)
PM Snack	5(1)	0(0)	2(0.2)	3(0.3)	23(1)	13(1)	17(1)	2(0.3)	9(1)	8(1)	2(0.4)	20(2)	32(3)	4(1)	1(0.2)	1(0.2)
Dinner	19(1)	1(0.1)	3(0.3)	5(0.4)	45(2)	17(1)	30(2)	1(0.2)	15(1)	4(1)	0(0)	12(1)	39(2)	3(0.4)	2(0.2)	1(0.2)
Evening Snack	13(1)	1(0.1)	2(0.2)	3(0.2)	15(1)	7(1)	8(1)	1(0.1)	6(0.3)	4(1)	1(0.2)	13(1)	42(3)	3(0.4)	2(0.2)	1(0.3)
test	<0.0001	<0.0001	<0.0001	<0.0001	<0.0001	<0.0001	<0.0001	<0.0001	<0.0001	<0.0001	<0.0001	<0.0001	<0.0001	<0.0001	<0.0001	<0.0001
